# Continuing education in healthcare as a strategy for occupational safety in the context of the COVID-19 pandemic: reflections on the role of community healthcare agents in construction of care

**DOI:** 10.47626/1679-4435-2021-669

**Published:** 2021-04-30

**Authors:** Gerardo Teixeira Azevedo Neto, Israel Coutinho Sampaio Lima, Ana Suelen Pedroza Cavalcante, Wallingson Michael Gonçalves Pereira, Maria Rocineide Ferreira da Silva, José Jackson Coelho Sampaio

**Affiliations:** 1 Atenção Primária à Saúde, Secretaria de Saúde, Irauçuba, CE, Brazil; 2 Programa de Pós-Graduação em Saúde Coletiva, Universidade Estadual do Ceará, Fortaleza, CE, Brazil

**Keywords:** community healthcare agents, continuing education in healthcare, occupational health, Primary Healthcare, COVID-19

## Abstract

Community healthcare agents are strategic professionals in delivery of Primary Healthcare activities. This article reflects on the role of continuing education in healthcare as a strategic element in ensuring the occupational health of community healthcare agents faced with combating and managing coronavirus 19 disease (COVID-19). In the current scenario, the work of community healthcare agents is undergoing daily reconstruction of professional practice in order to keep pace with the living territories to which they are assigned. Continuing education in healthcare enables construction of feasible scenarios that make problem solving possible, involving a constant reflective analytical perception of professional practices, permitting (re)construction of social skills such as the capacity to mobilize and motivate other actors to participate in political action. Through problematization, identification of needs, and questioning, continuing education in healthcare leads to (de)construction of the working practices of the very actors who are delivering care. Furthermore, continuing education in healthcare reaffirms the importance of the social, technical, and political training of community healthcare agents, thereby confirming their right to dignified and quality work. In a pandemic scenario, an agenda focused on continuing education in healthcare is essential to the continuity of care delivery to communities, facilitating expansion of access to the right to health.

## INTRODUCTION

The current global scenario of the severe public health crisis faced by the world’s healthcare systems as they attempt to combat the coronavirus 19 disease (COVID-19) is a hitherto unique phenomenon, never seen before this century. This situation has challenged the managers of economic, social, and healthcare sectors. The same is true in the context of community healthcare in Brazil, where community healthcare agents (CHA) are considered strategic professionals in delivery of Primary Healthcare Actions (PHA)^1^ targeting promotion, prevention, recovery, and rehabilitation of public health in the communities.

Community healthcare agents should be involved in constant processes of continuing education aimed at improving the service provided to their assigned areas and ensuring the quality of care delivery. However, since this is a new and unexpected situation, continuing education activities for coping with and managing COVID-19 in the context of community health need to be reflected on and planned in a manner that ensures these healthcare workers’ occupational health and safety. It will thus be possible to continue delivering primary healthcare to the Brazilian population.

In 2004, the Brazilian Health Ministry published Ordinance 198,^2^ making continuing education in healthcare (CEH) federal policy and, in 2007, Ordinance 1996^3^ set out the guidelines for implementation of this policy. Over the years, a need to update the policy was acknowledged, in response to the processes for financing and planning of activities at the state and municipal levels. As a result, in 2017, the Federal Government published Ordinance 3194, which created the Program for the Reinforcement of Continuing Education in Healthcare Practices in the Brazilian National Health Service (SUS - Sistema Único de Saúde), with the objective of stimulating, monitoring, and strengthening training of healthcare workers.^4^

Continuing education in healthcare is understood to be a collection of educational processes that are implemented to meet the learning needs of workers in their places of work, and which provide opportunities for reflection on their own processes, seeking to transform professional practices with the objective of improving them and guaranteeing the quality of the care provided, considering the power of teamwork.^5^ The underlying foundation is therefore the principles of the SUS applied to local requirements and collective analysis of working processes.^5^

Although application of CEH within PHA has achieved improvements in the level of professional training, the crisis triggered by COVID-19 tends to create doubts and anxieties related to methods of protection, to guidance, and to management of the disease in the community, particularly among the CHA, since they work in greatest proximity to the communities cared for as part of the Family Health Strategy (FHS). Community health agents can strengthen the ties between health professionals and the healthcare system’s clients.^6^ It is therefore necessary to understand and problematize the FHS’ activities starting from their working methods and the conditions in which they work in practice, in order to strengthen actions and strategies that contribute to coping with the pandemic or combating transmission of the disease.^7^

Considering this scenario, this article is justified by the importance of the CHA as PHA professionals who have knowledge of the local territory, access to the healthcare system, and the types of ties formed between healthcare teams and their clients, among other aspects. Additionally, they also conduct monitoring in the areas of greatest vulnerability, following-up suspected and milder cases. These professionals are therefore strategic to controlling the pandemic caused by COVID-19^8-9^ through health promotion and disease prevention. It is therefore of fundamental importance to engage in reflections that can generate strategies to ensure their occupational health in situations of risk, such as in the current scenario.

Furthermore, CEH strategies, as collective proposals, could give rise to appropriate solutions for the occupational health and safety of CHA, which should be developed and perfected with the objective of promoting options that can save these professionals’ lives. This study therefore raises the following question: on the basis of which elements should the processes for continuing education of CHA be considered and developed in the context of coping with and managing COVID-19, in order to guarantee their occupational health and safety, so that continuity of community primary healthcare can be guaranteed for the Brazilian population? The objective of this study is therefore to reflect upon continuing education in healthcare as a strategic element in guaranteeing the occupational health and safety of CHA in the context of coping with and managing COVID-19.

## METHODS

This is a theoretical paper presenting reflections on continuing education in healthcare as a strategic element in guaranteeing the occupational health and safety of CHA in the context of coping with and managing COVID-19. These reflections, presented below, are drawn from our practical experience as health professionals who provide training processes based on the learning needs of health professionals working in the many different settings of the SUS; our reflections in post-graduate public health classrooms; and our reading of articles and other important materials dealing with the team.

## RESULTS AND DISCUSSION

### THE CHALLENGES OF LEARNING IN THE CONTEXT OF COVID-19 PANDEMIC WITHIN THE WORKING SPECTRUM OF THE CHA

The CHAs’ work within the FHS teams has always been seen as of fundamental importance to delivery of primary healthcare activities. The relationships these professionals form with their peers within the community attempt to construct, in a problem-based manner, the means for health emancipation.^10^ These means can be important for fostering joint responsibility for care of the population based on their active role in the therapeutic plan that seeks the autonomy of health system clients without exempting health services of their responsibility.

Thus, the CHA have always been present in the homes of the FHS clients or in other community spaces. Home visits are a frequent part of their routine, helping the FHS in relation to territorialization, active searches, monitoring, and identification of the needs manifest by clients as a function of their health and the social, cultural, economic, and political needs of each area.^11^

Direct contact with people is a necessary part of implementing strategies for community health education through guidance on transmission of infectious agents, signs and symptoms, and the many different risks to health to which individuals and their families may be vulnerable, especially in situations of global emergency, as is the case of the current pandemic.^4,11^ Going beyond their actions in the community, the CHA daily support and strengthen access to and ties with the services provided by the FHS, disseminating information about the services offered by physicians, nurses, and dentists.^12^ However, as the crisis in Brazilian public health has been exacerbated by COVID-19, the CHAs’ work has been changing, following a pathway of reconstruction of professional practice.

In response to this process of reinvention, the Ministry of Health (MoH) published some proposals to guide the CHAs’ work in PHA settings in the context of the COVID-19 pandemic. These guidelines should be worked on by the entire FHS team and, as such, should be problematized and adapted to suit each region of the country by means of CEH.^13-14^ To achieve this, there is a constant process of creative invention of professional practices in healthcare and particularly within the FHS, since these are healthcare professionals who work in living and dynamic territories that are characterized not only by their geographic areas, but also by social relations, cohabiting arrangements that may create or diminish prospects for life and materialize the potentials and vulnerabilities of each area.^15^

These studies emphasize that although home visits are an important tool for actively seeking cases and monitoring suspected cases, it is important that CHA take certain precautions to protect their health and that of the recipients of their services, recoding their practices as a result. Recommended precautions that CHA should take when visiting clients include: not entering homes, conducting the contact in front of homes or in yards, outside, and prioritizing members of high-risk groups, such as people over the age of 60 or who have nontransmissible chronic diseases, i.e.: people with cardiac, respiratory, or renal diseases, those on immunosuppression and/or diabetics.^13-14^

Visits should be conducted observing a minimum distance of 1 meter and it is essential that the professionals wear surgical masks and the patients wear cloth masks. It is also essential to perform hand hygiene with soap and water or alcohol rub, and it is also necessary to guarantee that all other personal protective equipment (PPE) be supplied and worn, including, gloves, aprons, lab coats, goggles and/or acrylic face mask. Any CHA who has fever, dry cough, muscle pains, diarrhea, or other signs of sickness should be immediately isolated, to preserve both individual and collective health.^13-14^

One tactic for health promotion and saving lives while dealing with COVID-19 is the strategy of setting up groups on mobile apps, which is already being rolled out by the state Health Department of Rio de Janeiro. The CHA can set up groups on apps (WhatsApp for example) with residents of the areas they are assigned to, even when in social isolation. This facilitates sharing of correct information and guidance on the novel coronavirus, in addition to enabling monitoring of suspected patients, those at risk, or already detected and in home isolation, providing constant care by the entire FHS team.^16^

Additionally, any CHA over the age of 60 years and/or who have chronic conditions, such as those listed above, should be reassigned within the FHS to perform administrative tasks that do not require direct contact with the public.^13-14^ However, the varying real life situations put all of these MoH guidelines to the test, in their forms of learning and application. It is therefore recommended that each manager assesses their feasibility and applies the guidance according to the local situation and in dialog with the professionals who are providing care to the population.

Notwithstanding, situations are observed in which the CHA find themselves without support from their FHS teams and management in the context of the process of assimilation and reconstruction of the new working practices. As a result, their own health is risked and the activities for health promotion in the context of COVID-19 in the community are weakened.^10^

It is therefore necessary that that the FHS team and managers work in cohesion with the CHA, addressing their needs for improvements to and adaptation of their working practices to deal with COVID-19.^12^ It is necessary that written instructions be more didactic so that they can be easily assimilated and problematized in these professionals’ daily routines.

It is of fundamental importance that the whole FHS team supports the CHA, primarily the nurse, who is the coordinating agent of the team, according to the National Primary Care Policy.^17^ Cooperative mutual support among professionals will be capable of strengthening actions for combating and controlling COVID-19 and of preserving the health of the CHA and, as a consequence, of the population. It is thus essential to promote forms of conversation that instigate the CEH in order to foster learning about the way to use the different types of PPE, how to put them on, remove them, dispose of them, and/or sterilize them, avoiding self-contamination and contamination of others, among other subjects that may be raised, such as the training needed to improve the CHAs’ working practices.

### CEH AND ITS CONTRIBUTION TO THE OCCUPATIONAL HEALTH OF CHA IN THE CONTEXT OF COVID-19

In their daily work in the territories to which they are assigned, CHA professionals are exposed to multiple risks in the course of their duties.^18^ With the emergence of the pandemic,^19^ the entire healthcare community, as much as the CHA, must resignify the methods of achieving health promotion, caring for the population while caring for oneself.

In this manner, primary healthcare, which at the outset of the pandemic was “paralyzed” to a certain extent, can resume its services and activities with the objective of contributing to reducing transmission of COVID-19, thereby strengthening the FHS through the power of contact with families and communities in the living territory.^20^ Therefore, in order to venture into their assigned territories while adapting to the current situation in which we are living, the CHA must be in scrubs, with disposable aprons, surgical caps, face masks, gloves, and goggles. Thus equipped, they will be protected from the biological risks and from other risks to which they were already exposed in their working routines, such as physical and ergonomic risks.^21^ These risks have been amplified significantly by the emergence of the pandemic, primarily those related to contamination by microorganisms in droplets and/or aerossols.^22^

In this new scenario, it is possible that some errors will be committed in the processes of donning and doffing PPEs. These possible errors may be the primary causes of contamination of professionals who, initially, were performing their jobs remotely and have not previously had to wear these protective equipments every day.^23^

Within this field, the idea of multifunctional professionals is strong, and the CHA, in common with other professionals, multitask, which leads to overload, making them even more vulnerable to contamination.^6^ During the pandemic, this facet of healthcare work has been extended, since many professionals have had to increase their working hours to ensure effective care for the population.

Therefore, CEH is shown to be an important tool for ensuring these workers’ occupational safety. Problematization of working processes leads to identification of training needs that are important for decision-making. Questioning tends to generate solutions and reconstruction of working practices by the people who deliver the care process themselves.^24^ Thus, CEH makes it possible to construct feasible scenarios that make problem-solving possible.^25^

CEH can be conducted in informal spaces, during the working day of the employees working in the different health services and also in formal places of learning. During the pandemic, classrooms have also been modified and courses delivered via digital platforms also help to share knowledge. This ongoing process should involve a reflective analytical perception of working practices.^26^

Moreover, cooperation between health professionals can be a powerful ally of CEH in training professionals and transforming their healthcare practices. It is necessary to make professionals available to learn and to work in teams, building multiprofessional teams, collaborative working and learning groups that allow professionals to ensure the consistency of each other’s practices.^25^ This continuous reflection by the social actors working in the health services can foster CEH processes that should be capable of improving the provision of care, as can analysis of the territories in which these practices are delivered.^27^ CEH is therefore a strategic field of action for education and development of professionals that enables the (re)construction of social skills such as the capacity mobilize and motivate other actors to take part of a political action^28^ that is the agent of transformation of practices into a healthcare event performed in a constantly dynamic territory.

Actions of this nature help to reverse crystallization of hierarchical ideas of care and of in-service training processes caused by institutional longevity and repetition of protocols. From this springs the need to strengthen in-service dialog in order to ensure safer, more effective, and more efficient care. This activity, delivered collectively in communities, strengthens the service and the care provided by the SUS.^22^

At the same time as care is guaranteed within the principles of the SUS, the CHA have the opportunity to understand themselves better within their working processes, through analysis, criticism, and realignment of their exercises conducted during the course of CEH. This will make them more sensitive to the events that will continue to crop up *in loco*, giving them the support needed to solve them.^29^

## FINAL CONSIDERATIONS

CEH reaffirms the importance of social, technical, and political training of the CHA, providing them with the foundations to undertake work that guarantees their occupational health. Irrespective of the nature of the place of training, whether formal or informal, CEH represents a tool for training healthcare workers in the SUS. Thus, through identification of these workers’ training needs, using teaching and learning methodologies that promote learners’ critical thinking and autonomy and stimulate them to reflect on their working scenarios, the objective of CEH can be accomplished, i.e. working practices can be transformed.

It is by considering this process of transformation that society should readapt to face the current pandemic panorama. It is therefore necessary to encourage CEH activities through digital platforms that favor interprofessional communication, as participatory strategies inherent to the training processes and in accordance with the context in which each professional works. In this sense, a CEH-oriented agenda is essential in the pandemic scenario to ensure continuity of care provision in the community. Therefore, it is by reinventing their care that the CHA who work in situations of adversity can use CEH as an instrument to resignify their roles and continue to ensure expansion of access to the right to health.
